# A New Chimeric Natriuretic Peptide, C_N_AA_C_, for the Treatment of Left Ventricular Dysfunction after Myocardial Infarction

**DOI:** 10.1038/s41598-017-10748-6

**Published:** 2017-08-30

**Authors:** Shu-Miao Zhang, Hong-Lin Zhao, Xiao-Ming Gu, Juan Li, Na Feng, Yue-Min Wang, Rong Fan, Wen-Sheng Chen, Jian-Ming Pei

**Affiliations:** 1 Department of Physiology, National Key Discipline of Cell Biology, Fourth Military Medical University, Xi’an, 710032 Shaanxi Province China; 2 Department of Cardiovascular Surgery, the First Hospital of Lanzhou University, Lanzhou, 730030 Gansu Province China; 3 Department of Cardiovascular Surgery, Xijing Hospital, Fourth Military Medical University, Xi’an, Shaanxi Province China

## Abstract

An innovative natriuretic peptide analog named C_N_AA_C_ (structurally consisting of the C-terminus and ring of ANP and the N-terminus of CNP) that has been shown to exhibit potent vasodilatory, diuretic, and hypotensive effects in our previous study was evaluated for the treatment of left ventricular dysfunction following myocardial infarction. The temporal relaxation effect and metabolic status of C_N_AA_C_ were determined. A myocardial ischemic model was established. Rats were randomly divided into Sham, MI, MI-ANP, MI-CNP, MI-VNP, and MI-C_N_AA_C_ groups. Humoral factors were measured; echocardiography and hemodynamics methods were employed to assess the cardiac function at the fourth week after modeling. The results showed that C_N_AA_C_ had a potent relaxant effect and longer duration of action than ANP, CNP, or VNP. The stability of C_N_AA_C_ in blood was higher than other three NPs. Four weeks of NP administration ameliorated diastolic and systolic dysfunction, the hypertrophic index, myocardial fibrosis, and infarct size; it also restored the abnormal changes in humoral factors. These results demonstrate that C_N_AA_C_ has a potent cardioprotective effect against left ventricular dysfunction after myocardial infarction. The results may lay the foundation for the clinical application of this newly designed NP chimera in the treatment and prevention of heart failure.

## Introduction

Chronic heart failure is a clinical syndrome that is characterized by diastolic and systolic dysfunction and ejection function damage that result in an insufficient cardiac output that cannot fulfill the body’s needs. Chronic heart failure has also been associated with high morbidity and mortality^1^. The traditional treatment for heart failure is to strengthen cardiac function by administering several drugs, but the result is unsatisfactory. Recently, researchers realized that an adjustment of neurohormone activation is key in the treatment of heart failure. One of the adjustments is to restrain the function of positive regulation factors, such as the use of angiotensin-converting enzyme inhibitors (ACEI), to decrease the effects of angiotensin II and aldosterone, which will then alleviate the cardiac load and improve the coronary blood-supply. Another method is to strengthen the function of negative regulation factors and utilize the vasorelaxant, natriuretic, diuretic, and renin-angiotensin-aldosterone system (RAAS) inhibiting effects of natriuretic peptides to postpone the development of heart failure and myocardial remodeling^2^.

Natriuretic peptides (NPs) are a group of structurally similar but genetically distinct peptides that exert potent diuretic, natriuretic, vasorelaxant, antifibrotic, and antihypertrophic effects and play crucial roles in cardiovascular homeostasis^2, 3^. The study of natriuretic peptides began 50 years ago^4^. Earlier studies identified members of the NP family. After atrial natriuretic peptide (ANP) was found in atrial cells in the early 1980s, brain natriuretic peptide (BNP) and C-type natriuretic peptide (CNP) were identified in porcine brain between 1988 and 1990^5, 6^. *Dendroaspis* natriuretic peptide (DNP) was then identified in the venom of the green mamba in 1992^7^. Other peptides, including urodilatin and ventricle natriuretic peptide, were identified from the distal tubules of the kidney or ventricles of rainbow trout and eel and exhibited similar cardiovascular effects^8, 9^.

Although natriuretic peptides exert important effects in the cardiovascular system, the circulating half-life of ANP is only 3–5 min, the half-life of BNP is approximately 23 min^4^, and the half-life of CNP is only 2–3 min. Although designed-ANP and BNP analogs have been widely used to treat experimental heart failure, the clinical application of natriuretic peptides has been limited^10, 11^ due to the low chemical synthetic efficiency, expensive fabrication cost, and vulnerability to degradation *in vivo*.

In recent studies, synthetic chimeric natriuretic peptides, such as VNP, CD-NP, and CU-NP, were shown to maximize the beneficial properties of their donor natriuretic peptides^12–15^, and the extension of natural natriuretic peptide applications has become a research hotspot. In our previous study, we innovatively designed and synthesized a chimera peptide named C_N_AA_C_^16^ (the C-terminus and ring of ANP was combined with the N-terminus of CNP), which has potent vasorelaxant, diuretic and hypotensive effects. These actions of C_N_AA_C_ imply that it may be applicable in the treatment of heart failure.

Therefore, the present study was designed to determine whether C_N_AA_C_ has a longer duration of action and higher stability than ANP, CNP or VNP and whether it can be used to treat left ventricular dysfunction after myocardial infarction in rats.

## Results

### Temporal Course of Natriuretic Peptide-Induced Relaxation

**Figure 1:**
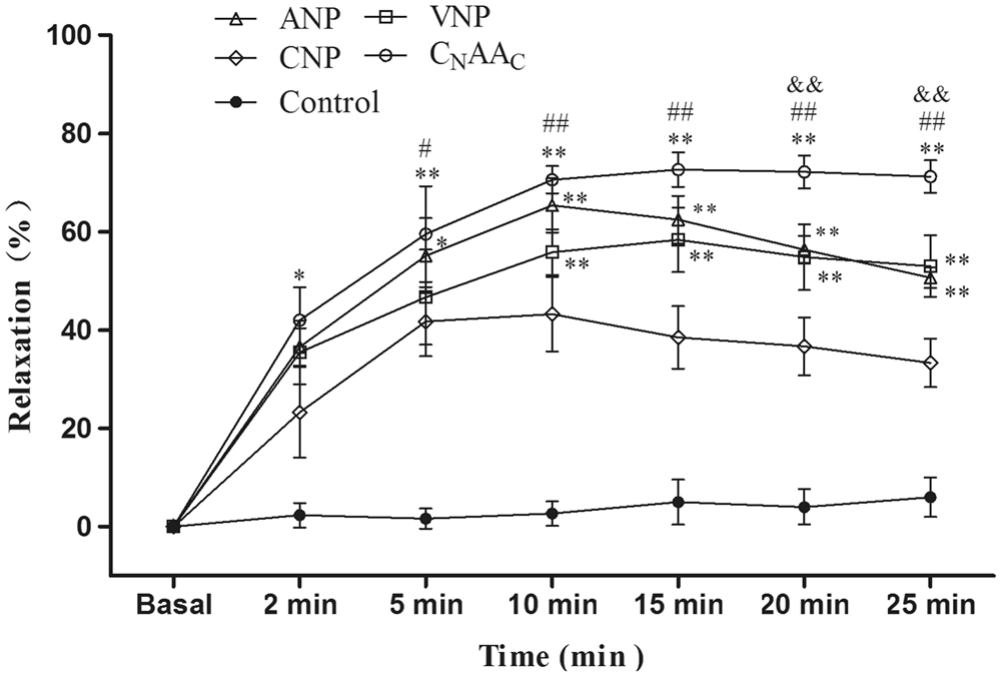
Temporal course of relaxation induced by NPs at a single-dose of 10^−6^ M. Data are expressed as the mean ± the SEM; n = 6 in each experimental group. *P < 0.05, **P < 0.01 vs. CNP; ^#^ *P* < 0.05, ^##^ *P* < 0.01 vs. VNP; ^&&^ *P* < 0.01 vs. ANP.

Figure 1 illustrates the effect of a single concentration (10^−6^ M) of NPs on NE-induced contractility. Control strips were steady during the observation time and were not significantly different from the basal level. The relaxation induced by ANP or CNP decreased after 10 min, relaxation induced by VNP decreased after 15 min, and relaxation induced by C_N_AA_C_ increased steadily over the entire 25 min. Relaxation in response to all the peptides remained significantly greater than the basal level throughout the 25-min study period (*P* < 0.01).

During the first 5 min, the magnitude of the relaxation induced by C_N_AA_C_ or ANP was greater than that produced by CNP (*P* < 0.05). During 10, 15, 20, and 25 min, the magnitude of the relaxation induced by C_N_AA_C_, VNP, or ANP was greater than that induced by CNP (*P* < 0.01). The relaxation effect of C_N_AA_C_ was greater than that of VNP at 10, 15, 20, and 25 min (*P* < 0.01) and was also greater than ANP at 20 and 25 min (*P* < 0.01).

### Stability of C_N_AA_C_ in the Circulation of Rats

To further investigate the stability of C_N_AA_C_, NPs were administered, the rat serum was separated, and the remaining NPs were detected.

**Figure 2:**
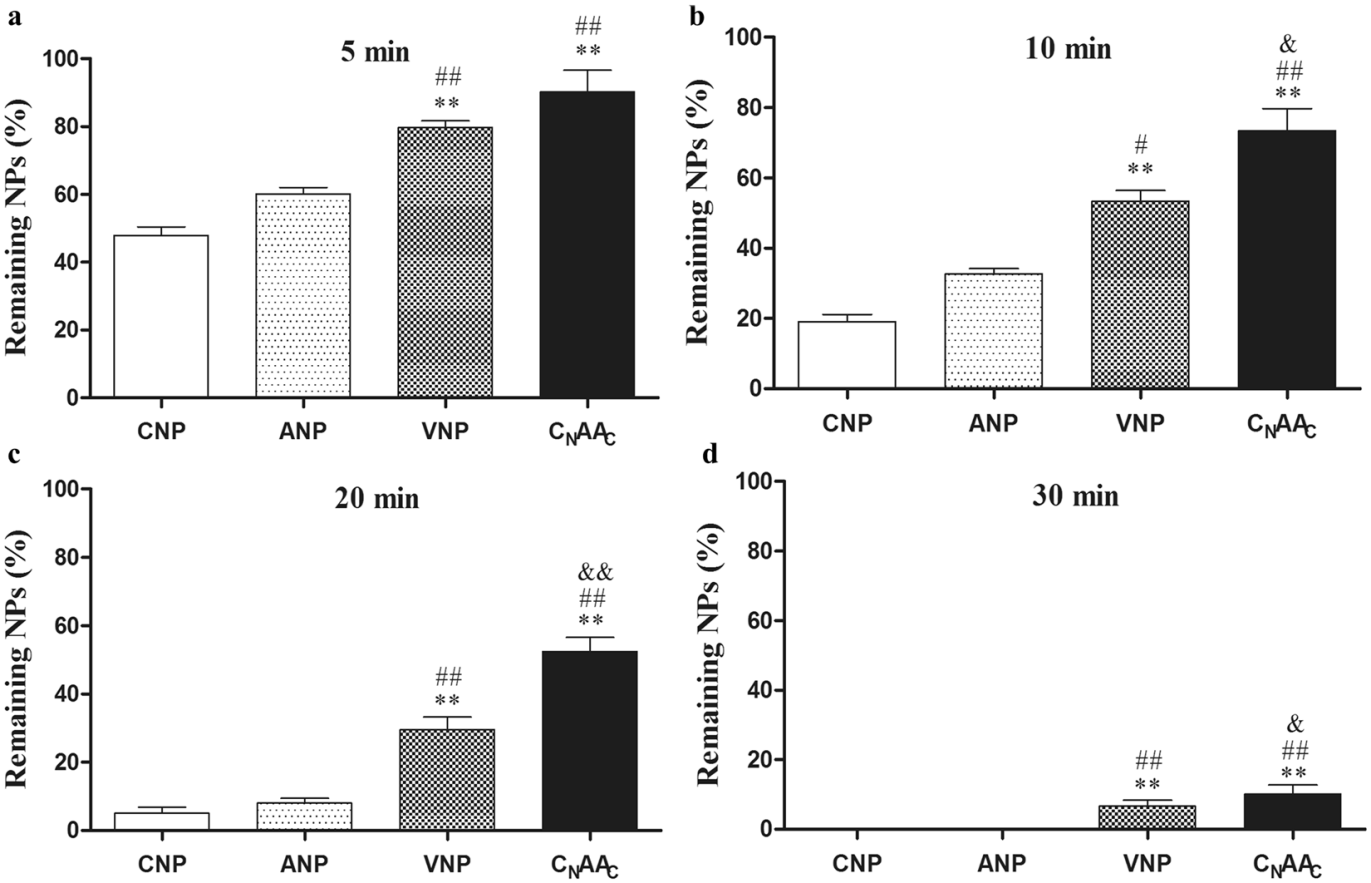
Degradation of NPs in the blood of rats after a bolus injection. Data are expressed as the mean ± the SEM; n = 5 in each experimental group. **P < 0.01 vs. CNP; ^##^ *P* < 0.01 vs. ANP; ^&^ *P* < 0.05, ^&&^ *P* < 0.01 vs. VNP.

In the first 5 min, 52.2% of CNP, 39.9% of ANP, 20.3% of VNP, and 9.9% of C_N_AA_C_ were degraded (Fig. 2a). The remaining levels of VNP and C_N_AA_C_ were greater than those of ANP or CNP (*P* < 0.01). At 10 min, only 19.1% of CNP and 32.7% of ANP remained, and the remaining VNP and C_N_AA_C_ levels were higher than those of ANP or CNP (*P* < 0.05 or *P* < 0.01) (Fig. 2b); the remaining level of C_N_AA_C_ was greater than that of VNP (*P* < 0.05). Less than 10% of ANP or CNP remained at 20 min (Fig. 2c), and the compounds were completely degraded at 30 min. More than 30% of VNP and C_N_AA_C_ remained at 20 min, the remaining level of _CN_AA_C_ was greater than that of VNP (*P* < 0.01) at 20 min. At 30 min, the remaining VNP and C_N_AA_C_ levels were still detectable, the remaining level of _CN_AA_C_ was greater than VNP (*P* < 0.05) (Fig. 2d).

### Effect of C_N_AA_C_ on Cardiac Hypertrophy after Myocardial Infarction

After 4 weeks, body weights were decreased in the MI group compared to the sham group (*P* < 0.05) and lung weights were similar among groups (Table 1). The left ventricular weight, heart weight, ratios of the left ventricular weight to body weight (LVW/BW), heart weight to body weight (HW/BW), and lung weight to body weight (LW/BW) in the MI group were higher than in the sham group (*P* < 0.01). The MI-NP groups showed significantly reduced LVW/BW and HW/BW compared with the MI group (*P* < 0.05 or *P* < 0.01), and only the C_N_AA_C_-treated group showed significantly reduced LW/BW (*P* < 0.05).

**Table 1:** Effects of NPs on tissue weights after myocardial infarction.

Parameters	Sham	MI	MI-ANP	MI-CNP	MI-VNP	MI-C_N_AA_C_
BW, g	380 ± 9	338 ± 10*	362 ± 8	347 ± 10	337 ± 16	352 ± 11
LVW, mg	346 ± 22	570 ± 27**	492 ± 24	492 ± 24	485 ± 26	433 ± 15^†^
HW, mg	976 ± 43	1138 ± 37*	1010 ± 39	1072 ± 84	998 ± 48	980 ± 26^†^
LW, mg	1764 ± 164	1890 ± 137	2094 ± 188	2190 ± 149	1960 ± 221	2214 ± 284
LVW/BW, mg/g	1.12 ± 0.05	1.76 ± 0.08**	1.37 ± 0.07^††^	1.31 ± 0.05^††^	1.40 ± 0.08^††^	1.29 ± 0.03^††^
HW/BW, mg/g	2.50 ± 0.05	3.46 ± 0.10**	2.69 ± 0.07^††^	3.03 ± 0.08^†^	2.8 ± 0.11^††^	2.90 ± 0.13^††^
LW/BW, mg/g	4.33 ± 0.18	6.00 ± 0.29**	5.21 ± 0.41	5.30 ± 0.23	4.79 ± 0.26	4.56 ± 0.17^†^

MI: myocardial infarction, BW: body weight, LVW: left ventricular weight, HW: heart weight, LV: left ventricle, LW: lung weight, *P < 0.05, **P < 0.01 vs. Sham; ^†^ *P* < 0.05, ^††^ *P* < 0.01 vs. MI. Results are presented as the mean ± the SEM; n = 6 in each experimental group.

### Effect of C_N_AA_C_ on Hemodynamics and Left Ventricular Remodeling

Hemodynamics examinations revealed that the heart rate did not change in each group, LVSP and ± dp/dt_max_ were significantly decreased (*P* < 0.01) (Table 2), and LVEDP was significantly increased in the MI group (*P* < 0.01). After NP administration, left ventricular function, as reflected by these parameters, was reversed (*P* < 0.05, *P* < 0.01) (Table 2). These results suggest that natural NP and C_N_AA_C_ elicited a significant protective effect on cardiac function.

**Table 2:** Effects of NPs on cardiac hemodynamics after myocardial infarction.

Groups	Heart rate, (bpm)	LVSP (mm Hg)	LVEDP (mm Hg)	+dp/dt_max_	−dp/dt_max_
Sham	457 ± 18	139.8 ± 2.3	5.8 ± 1.2	4341 ± 693	4302 ± 520
MI	421 ± 22	96.8 ± 5.4**	10.8 ± 1.6**	2924 ± 348**	2853 ± 395**
MI-ANP	437 ± 15	112.5 ± 3.9^††^	7.2 ± 1.8^†^	3651 ± 320^††^	3424 ± 453^††^
MI-CNP	443 ± 21	122.7 ± 4.8^†^	6.5 ± 1.5^††^	4025 ± 289^††^	3917 ± 250^††^
MI-VNP	430 ± 20	119.3 ± 5.6^††^	7.0 ± 1.3^††^	4178 ± 451^††^	3984 ± 527^††^
MI-C_N_AA_C_	432 ± 29	127.5 ± 4.2^††^	6.8 ± 1.1^††^	4214 ± 161^††^	4025 ± 199^††^

**P < 0.01 vs. Sham; ^†^ *P* < 0.05, ^††^ *P* < 0.01 vs. MI. Results are presented as the mean ± the SEM; n = 6 in each experimental group.

**Figure 3:**
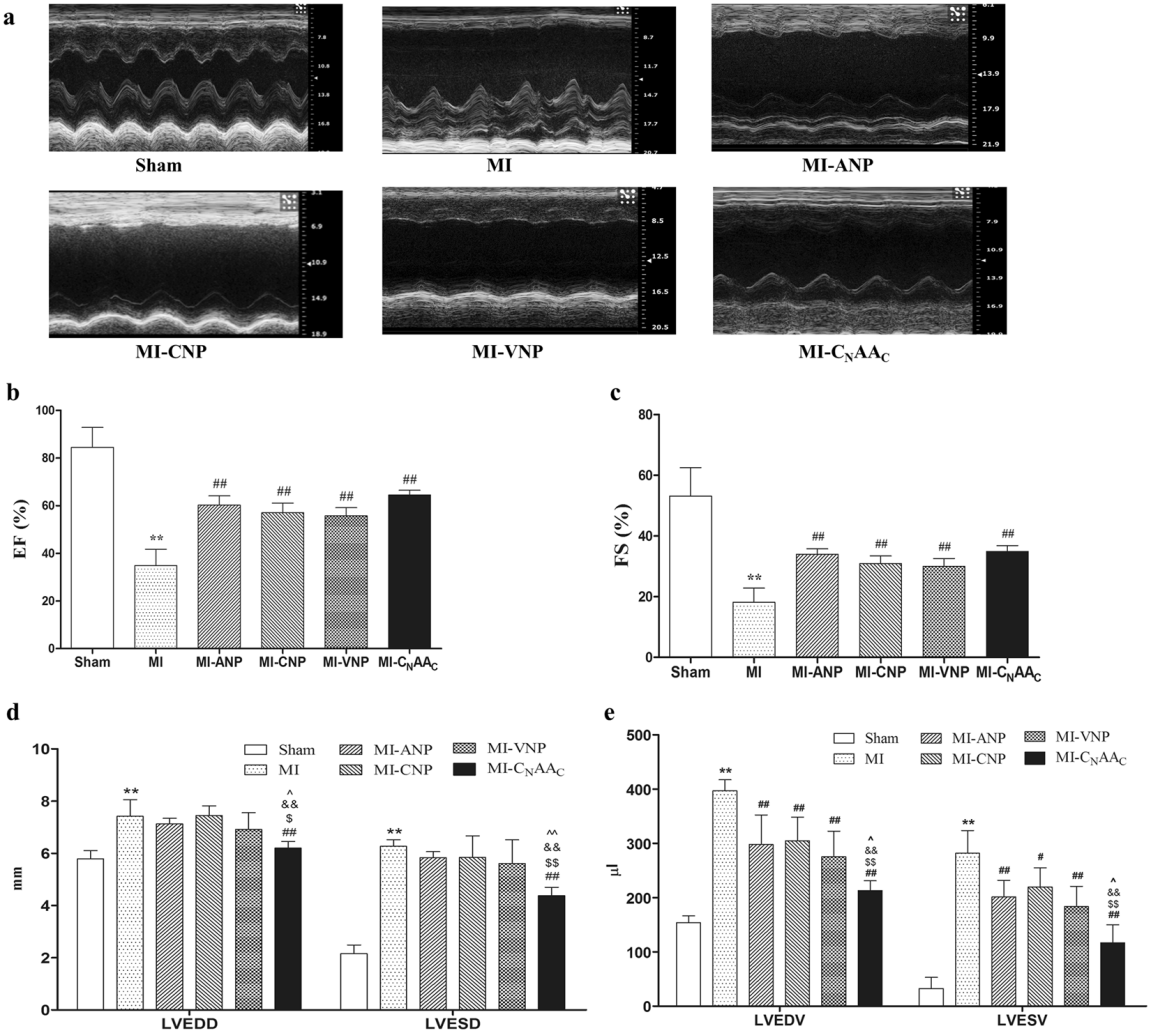
Effects of NPs on left ventricular remodeling after myocardial infarction. **(a)** Echocardiography in each group. (**b,c,d**) Effects of NPs on cardiac function (n = 6). EF: Left ventricular ejection fraction, FS: Left ventricular fractional shortening, LVEDD: left ventricular end diastolic diameter, LVESD: left ventricular end systolic diameter, LVEDV: left ventricular end-diastolic volume, LVESV: left ventricular end-systolic volume. Data are expressed as the mean ± the SEM. **P < 0.01 vs. Sham, ^##^ *P* < 0.01 vs. MI, ^$^ *P* < 0.05, ^$$^ *P* < 0.01 vs. MI-ANP, ^&&^ *P* < 0.01 vs. MI-CNP, vs. ^^^ *P* < 0.05, ^^^^ *P* < 0.01 vs. MI-VNP.

After 4 weeks, the impulses of the ventricular anterior wall in the MI group weakened significantly and the heart volume enlarged, which were detected by echocardiography (Fig. 3a); cardiac function also improved in the NP-treated groups. Compared with the Sham group, EF and FS in the MI group decreased significantly (*P* < 0.01) (Fig. 3b,c) and the LVEDD, LVESD, LVEDV, and LVESV increased significantly (*P* < 0.01) (Fig. 3d,e). In addition, all the MI-NPs groups showed elevated EF and FS (*P* < 0.01). Only the MI-C_N_AA_C_ group showed decreased LVEDD and LVESD compared with the MI group (*P* < 0.01), and the effect of C_N_AA_C_ was stronger than the other three NPs (*P* < 0_._05 or *P* < 0.01) (Fig. 3d). LVEDV and LVESV were decreased in all four NP groups (*P* < 0.01), and the effect of C_N_AA_C_ was more potent than the other N*P*s (*P* < 0.05 or *P* < 0.01) (Fig. 3e).

### Examination of the Infarct Size and Collage Volume Fraction after Myocardial Infarction

**Figure 4:**
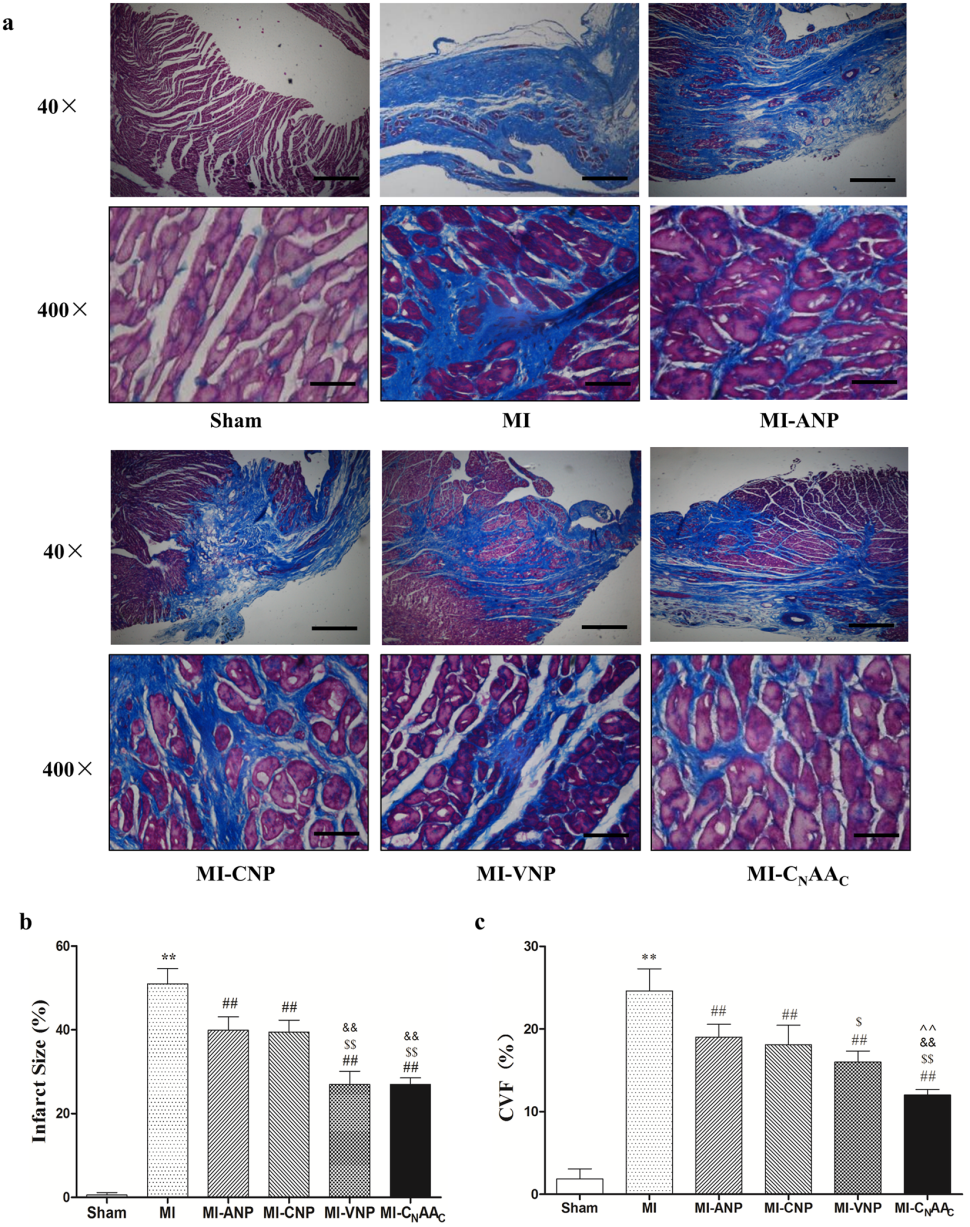
Effect of NPs on myocardial infarct size and collagen volume fraction. (**a)** Representative images of Masson’s trichrome stained blue collagen in each group (original magnification, 40× , 400× ). The scale bars in 40 × magnification pictures represent 200 μm, the scale bars in 400× magnification pictures represent 20 μm. The cytoplasm appears dark-red and collagen fibers are blue; (**b)** Effect of NPs on myocardial infarct size; (**c**) Effect of NPs on collagen volume fraction. Gross size in the MI group was larger than that in the Sham group. Wall thinning and the whitish color of the infarcted areas in left ventricle were detected with Masson’s trichrome stain, and thick blue colored fibrosis was found (original magnification, 400× ). CVF: collagen volume fraction. Data are expressed as the mean ± the SEM; n = 5 in each experimental group. **P < 0.01 vs. Sham; ^##^ *P* < 0.01 vs. MI; ^$^ *P* < 0.05, ^$$^ *P* < 0.01 vs. MI-ANP; ^&&^ *P* < 0.01 vs. MI-CNP; ^^^^ *P* < 0.01 vs. MI-VNP.

As shown in Masson’s trichrome stain (optical microscopy), the MI group showed a diffused brick-red staining in the cytoplasm, with large scales of blue collagen, and less blue collagen was observed in the MI-NP groups (Masson’s trichrome stain, 40 × , 400 × , Fig. 4a). Compared with the Sham group, the infarct size and collage volume fraction (CVF) in the MI group were significantly increased (*P* < 0.01) (Fig. 4b,c). The infarct size and CVF decreased significantly after NP administration (*P* < 0.01). The effects of VNP and C_N_AA_C_ on the infarct size were stronger than those of ANP or CNP (*P* < 0.01). The effect of VNP on CVF was stronger than that of ANP, and the effect of C_N_AA_C_ was stronger than other three NPs (*P* < 0.01) (Fig. 4c).

### Examination of Humoral Factors in Serum

**Figure 5:**
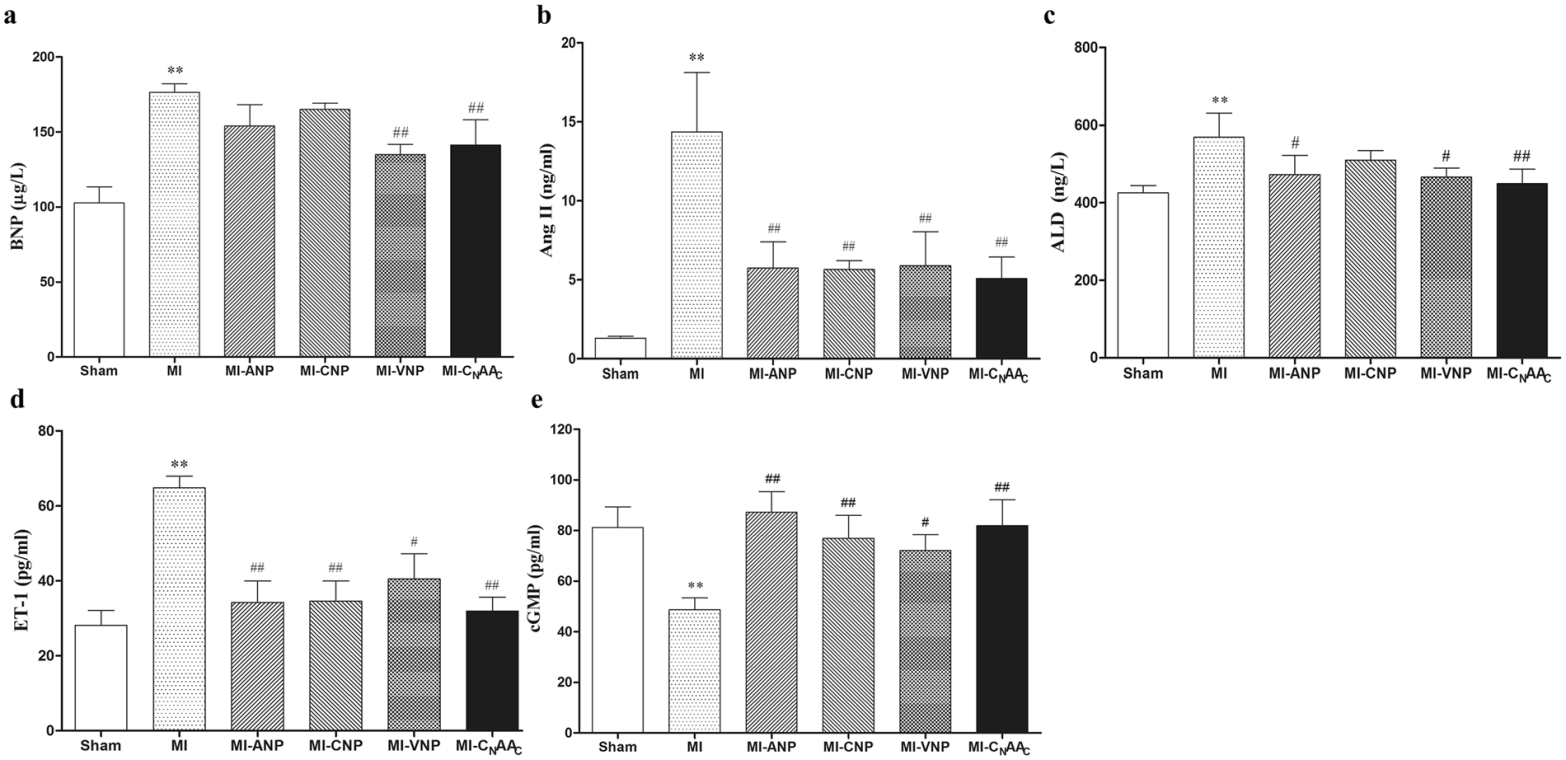
Effect of NPs on humoral factors in the serum of rats. **(a)** Effect of NPs on serum BNP levels in rats; (**b)** Effect of NPs on serum Ang II levels in rats; (**c)** Effect of NPs on plasma ALD levels in rats; (**d)** Effect of NPs on serum ET-1 levels in rats; (**e**) Effect of NPs on serum cGMP levels in rats (n = 6). Data are expressed as the mean ± the SEM; n = 6 in each experimental group. **P < 0.01 vs. Sham; ^#^ *P* < 0.05, ^##^ *P* < 0.01 vs. MI.

At 4 weeks after surgery, the serum BNP, Ang II, ALD and ET-1 levels in the MI group increased significantly compared with those of the sham group (*P* < 0.01) (Fig. 5). The serum level of BNP decreased only after VNP or C_N_AA_C_ administration (*P* < 0.01) (Fig. 5a); the serum level of Ang II and ET-1 decreased after ANP, CNP, VNP or C_N_AA_C_ administration (*P* < 0.05 or *P* < 0.01) (Fig. 5b and d); and the serum level of ALD decreased after ANP, VNP, and C_N_AA_C_ administration (*P* < 0.05 or *P* < 0.01) (Fig. 5c). The serum level of cGMP in the MI group decreased dramatically compared with that in the sham group, whereas it was increased after ANP, CNP, VNP or C_N_AA_C_ administration (*P* < 0.05 or *P* < 0.01) (Fig. 5e).

## Discussion

In our previous study, we confirmed that the new chimera C_N_AA_C_ had potent vasorelaxant, diuresis and hypotensive effects. Based on these cardiorenal effects, we further demonstrated that C_N_AA_C_ elicited inhibitory effects on left ventricular remodeling in the present study. A continuous infusion of C_N_AA_C_ for four weeks improved the systolic and diastolic function, ameliorated myocardial infarction and fibrosis, and even normalized plasma biomarkers of heart failure.

Our previous study demonstrated that C_N_AA_C_ had more potent vasorelaxant effects than ANP and CNP^16^. To better understand whether C_N_AA_C_ had a longer effect time than the other NPs, the abdominal aorta was obtained and a temporal examination was performed. We found that the vasorelaxant effect of C_N_AA_C_ lasted for a long time and steadily increased over the entire examination. By contrast, the relaxation induced by ANP or CNP began to decrease at 10 min, and the relaxation induced by VNP began to decrease at 15 min. The results suggested that C_N_AA_C_ has a longer duration of action than ANP, CNP, or VNP. To further confirm the stability of C_N_AA_C_ in circulation, the remaining NPs in the serum were detected after NPs were administered. The remaining NPs were detected at 5, 10, 20, and 30 min. It has been reported that the half-life of ANP and CNP is very short, almost 2–3 min^4^. The results of the present study are in accordance with previous results. The levels of CNP and ANP decreased by almost half in the first 5 min, while the levels of VNP and C_N_AA_C_ were stable. Over time, VNP and C_N_AA_C_ were degraded slowly, and at 30 min, some VNP and C_N_AA_C_ were still present. This result suggests that chimeric natriuretic peptides are stable in circulation and have longer durations of action than natural natriuretic peptides.

Cardiac dysfunction and pathophysiologic adaption were observed in rats subjected to ligation of the coronary artery^17, 18^. In the current study, the left ventricular dysfunction model was established via ligation of the coronary artery for 4 weeks. After 4 weeks, MI rats showed increased LVW/BW, HW/BW, and LW/BW, indicating that MI rats exhibited severely cardiac hypertrophy and pulmonary congestion; these results were consistent with others^19^. The four MI-NP groups showed reduced LVW/BW and HW/BW, and the C_N_AA_C_-treated group showed reduced left ventricular weight, heart weight and LW/BW, indicating a satisfactory reversal of cardiac hypertrophy.

Diastolic dysfunction caused by coronary artery ligation always appears earlier than systolic dysfunction^20^. MI rats displayed increased LVEDP, LVEDD, LVESD, LVEDV and LVESV and decreased LVSP, ± dp/dt_max_, EF and FS; all these changes reflect serious diastolic and systolic dysfunction of the left ventricle in rats. Many studies have demonstrated the effect of NPs and their analogs in the treatment of myocardial ischemia and infarction^15, 21–23^. Our data showed that a 4-week intraperitoneal injection of natriuretic peptides (20 nmol/kg/2 d) apparently ameliorates both cardiac systolic (LVSP, EF, FS, + dp/dt_max_) and diastolic function (LVEDP, −dp/dt_max_). In particular, C_N_AA_C_ significantly decreased LVEDD, LVESD, LVEDV, and LVESV, and these effects were greater than with the other three natriuretic peptides, suggesting that C_N_AA_C_ significantly improves cardiac function and the effect of C_N_AA_C_ is more potent than its donor or homology natriuretic peptide in some respects.

C_N_AA_C_ has more potent relaxation effect than ANP, CNP, and VNP, thus our previous study showed that C_N_AA_C_ has more potent hypotensive and diuretic effects in normal rats^16^. The peripheral vascular resistance (after-loading) and blood volume were reduced due to the vasodilation and diuresis caused by C_N_AA_C_, therefore C_N_AA_C_ exerts potent cardioprotective effect in the present study, and shows more efficacious than other peptides in some parameters. Because C_N_AA_C_ has more potent vasorelaxant effect, the venous return in the MI-C_N_AA_C_ group was reduced relatively compare with other NP-treated groups. On the other hand, MI-C_N_AA_C_ group showed more potent effect to reduce the LVEDV and LVESV, it means the myocardial contractility was improved in the MI-C_N_AA_C_ group. Combine these two aspects, the EF may remain unchanged compared with other NP-treated groups.

Histological analysis demonstrated that myocardial fibrosis and infarct size were significantly decreased after administration of the four natriuretic peptides (i.p. for 4 weeks). The effect of VNP or C_N_AA_C_ was greater than that of ANP or CNP. These results suggest that C_N_AA_C_ may have a potent cardioprotective effect.

In the present study, besides consulting the typical operation method from other researchers^24, 25^, operation was conducted by same laboratory technician, ligation was carried out at the same position of coronary artery, and the same age and weight of rats were adopted, and above all, the survival rats which undergone surgery were randomly divided into MI or MI-NP groups. All these steps should objectively ensure that there are no significant differences among groups before NPs treatment. However, very recent study has reported that serum concentration of troponin I should be measured 24 h after coronary artery ligation to indirectly estimate myocardial infarct size and confirm that there are no differences among groups^26^. We totally agree that this is really a more effective method to avoid between-group variance and is valuable to be used in the future study.

The level of serum BNP has been shown to be elevated in patients with left ventricular dysfunction^27^, and has been widely used in the diagnosis of heart failure^11^. It was demonstrated in the present study that the BNP level was decreased after VNP or C_N_AA_C_ was administered. The potential reason for this decrease is that the cardiac load was decreased and cardiac function was improved by VNP or C_N_AA_C_. In addition, the effect of precipitating factors that cause BNP release from the heart was decreased by C_N_AA_C_ administration. RAAS has been shown to be activated in rats after myocardial infarction, and the serum Ang II and ALD levels were shown to be significantly increased in the MI group in the present study. Previous studies have shown that plasma ALD levels may be elevated significantly in HF patients, primarily due to an increased production by the adrenal glands following stimulation by the high plasma Ang II concentrations^28, 29^. Although ANP and BNP were released from the heart after MI, the amount was insufficient to resist the effect of the sympathetic nervous system, RAAS, and vasopressin. RAAS inhibitors are currently the cornerstone pharmacotherapy for chronic heart failure^30^, and the present study showed that the Ang II and ALD levels were significantly decreased by C_N_AA_C_. These results suggested that RAAS, a precipitating factor that causes BNP release from the heart, was inhibited by C_N_AA_C_.

ET-1 participates in the pathological process of heart failure or myocardial infarction^31^. Jandeleit-Dahm KA *et al*.^20, 32, 33^ found that natriuretic peptides promote cGMP production to inhibit the ET-1 synthesis caused by ischemia or hypoxia. In the present study, when rats were subjected to myocardial infarction, the serum ET-1 level increased significantly, and this elevated ET-1 was decreased by treatment with natriuretic peptides. In our previous study, abdominal aortic cGMP production was increased after incubation with natriuretic peptides; in the present study, serum cGMP was decreased in the MI group and the cGMP level was increased after natriuretic peptide administration, which are in accordance with previous studies^34, 35^.

Taken together, our results demonstrate that C_N_AA_C_, a newly designed NP chimera, exerts a longer duration of action and potent cardioprotective effects against left ventricular dysfunction. These findings may lay the foundation for future studies aimed at the clinical application of natriuretic peptide chimeras in the treatment and prevention of heart failure after myocardial infarction.

## Methods

### Peptide synthesis and Reagents

Human ANP, CNP, VNP and the designed C_N_AA_C_ were synthesized by GL Biochem Ltd. (Shanghai, China) and were confirmed to have a purity of approximately 95% by high-performance liquid chromatography analysis. ACh and L-norepinephrine (NE) were purchased from Sigma-Aldrich Co. LLC (St. Louis, MO, USA). Rat BNP, angiotensin II (Ang II), aldosterone (ALD), endothelin-1 (ET-1), and cyclic guanosine monophosphate (cGMP) ELISA kits were purchased from Shanghai Lianshuo Biological Technology Co., Ltd. (Lianshuo, Shanghai, China).

### Animals

Male Sprague-Dawley (SD) rats (250 ± 20 g body weight) from the animal center of the Fourth Military Medical University were housed under specific pathogen-free conditions, with free access to water and food and a 12 h light/12 h dark cycle. All experiments were conducted according to *the Guide for the Care and Use of Laboratory Animals* published by the US National Institutes of Health (NIH Publication No. 85–23, revised 1985) and were approved by University Ethics (the Ethical Committee of the Fourth Military Medical University).

Although we identified that C_N_AA_C_ has potent vasodilating, diuretic, and hypotensive effects, it was not clear whether it has a longer duration of action than its analog VNP or other natural NPs. Therefore, *in vivo* and *in vitro* experiments were conducted as follows.

### Examination of the temporal relaxation effect of C_N_AA_C_

Rats were sacrificed and the abdominal aorta was obtained. The abdominal aorta, K-H solution, experimentation system, and the operational program were prepared and used as described in our previous study^12^. After a 30-min equilibration, NE (10^−6^ M) was added to induce a vasoconstriction effect. The contraction amplitude caused by NE (10^−6^ M) is approximately 1750 ± 250 mg in the control and the treatment group. To study the temporal relaxation effect of C_N_AA_C_, Normal Saline or NPs (10^−6^ M) were added when a stable contraction was reached. The effect of NPs on abdominal aorta contractility was measured as the activity during the 5-min period after their administration to the organ bath, and the data were recorded in 5-min intervals. Each maximal contraction induced by 10^−6^ M NE was arbitrarily set as 100%. Relaxation data are the reduced percentage of maximal contractile amplitudes^16^.

### Examination of the metabolic status of C_N_AA_C_

To determine the metabolic status of NPs in circulation, blood was collected from rats after a bolus injection of NPs. Rats were anesthetized with pentobarbital sodium (40 mg/kg i.p). One microcatheter was inserted into the left jugular vein to administer NPs, and another one was inserted into the right carotid artery to collect blood. After the surgery was completed, rats were allowed to stabilize for 15 min. Human ANP, CNP, VNP, or C_N_AA_C_ at a concentration of 40 nmol/kg was administered in a bolus fashion (0.1 ml). Blood (0.5 ml) was sampled 5, 10, 20, and 30 min after the bolus injection. The samples from the plasma were analyzed using LT-20AT High-Performance-Liquid-Chromatography (HPLC) (Shimadzu, Tokyo, Japan). A linear gradient (Buffer A: acetonitrile with 0.05% trifluoroacetic acid; Buffer B: double distilled water with 0.05% trifluoroacetic acid)^36^ was used for both analyses to separate peptides on an Inertsil OSD-3 (250 mm × 4.6 mm, 5 µm) chromatographic column (Shimadzu, Tokyo, Japan) and a Water Quatro-premier tandem quadrupole mass spectrometry and Alliance 2695 chromatographic system (Waters, Milford, Massachusetts, USA). Peak areas have been calculated with the Masslynx 4.1 software from Waters. Four independent experiments were performed. Each experiment was conducted in duplicate, whereby duplicates are defined as two different vials.

### Examination of the cardioprotective effect of C_N_AA_C_

Animals were divided into six groups: Group 1: Sham (i.p. 0.5 ml 0.85% NaCl/2d; n = 6); Group 2: MI (i.p. 0.5 ml 0.85% NaCl/2d; n = 6); Group 3: MI-ANP (i.p. 0.5 ml ANP, 20 nmol/kg·2d; n = 6); Group 4: MI-CNP (i.p. 0.5 ml CNP, 20 nmol/kg·2d; n = 6); Group 5: MI-VNP (i.p. 0.5 ml VNP, 20 nmol/kg·2d; n = 6); Group 6: MI-C_N_AA_C_ (i.p. 0.85% NaCl; 20 nmol/kg·2d; n = 6).

The Sham and MI animal model were established as described by Johns TN *et al*.^24, 25^ Rats were anesthetized with pentobarbital sodium (40 mg/kg i.p.), and then the left anterior descending coronary artery was ligated in the MI and MI-NP groups. The chest was closed immediately. Only a thoracotomy and closure were performed in the sham group. From the day of surgery, 0.85% NaCl or NPs (20 nmol/kg) were administered to these groups (i.p), one injection every two days and lasted for four weeks. Four weeks later, the cardiac function was measured by Doppler echocardiography under anesthesia with pentobarbital sodium (40 mg/kg i.p.). A 12 MHz linear probe (Vevo2100, VisualSonics, Inc., CAN) was used to perform the transthoracic echocardiography. Left ventricular (LV) ejection fraction (EF), LV fraction shortening (FS), LV end diastolic diameter (LVEDd), and left ventricular end systolic diameter (LVESd) were measured. All examinations were performed in a blinded state.

The day after echocardiography was performed; rats in each group were weighed and anesthetized via a peritoneal injection of pentobarbital sodium (40 mg/kg i.p.). Supplemental doses of sodium pentobarbital were given when needed to maintain uniform anesthesia. A micro catheter was inserted into the left ventricle through the right external jugular artery, and left ventricular systolic pressure (LVSP), left ventricular end diastolic pressure (LVEDP), and ± dp/dt_max_ were recorded by the BL-420F BioData Acquisition & Analysis Systems (TME Technology Co, Ltd, Chengdu, China). At the end of the experiment, blood was sampled and the following parameters were recorded: heart weight (HW), left ventricle weight (LVW), and lung weight (LW).

### Analysis of humoral factors

Blood samples were centrifuged (4000 rpm, 15 min) and the serum was separated. Serum BNP, Ang II, ALD, ET-1, cGMP levels were detected with rat ELISA kits (Lianshuo, Shanghai, China).

### Histological analysis

Rats in each group were anesthetized with pentobarbital sodium (40 mg/kg i.p.). Their hearts were then separated and fixed in 4% formaldehyde for 48 hours, and Masson’s trichrome stain was performed. Myocardial infarction and fibrosis were observed by Masson staining. We calculated infarct size as follows: Infarct size (%) = Length of infarction area / (inner perimeter of left ventricle + outer perimeter of left ventricle) /2 × 100%. We randomly chose six segments, and then photomicrographs of each segment stained by Masson trichrome were acquired with a scanner. Imagin-Pro Plus (version 6.0) was used to calculate the collagen volume fraction (CVF): CVF (%) = collagen area / total area × 100%.

### Statistical analysis

All data were expressed as the mean ± the SEM and analyzed with either t-tests (two groups) or ANOVAs (three or more groups). A Bonferroni correction was performed for post hoc *t*-tests. *P* values less than 0.05 were considered statistically significant. Graphpad prism 5 was used to create all artwork.
